# Assessment of Type 2 Diabetes Risk in Young Women with Polycystic Ovary Syndrome

**DOI:** 10.3390/diagnostics13122067

**Published:** 2023-06-14

**Authors:** Sarantis Livadas, Rodis Paparodis, Panagiotis Anagnostis, Alessandra Gambineri, Jelica Bjekić-Macut, Tijana Petrović, Bulent O. Yildiz, Dragan Micić, George Mastorakos, Djuro Macut

**Affiliations:** 1 Endocrine Unit, Athens Medical Centre, 65403 Athens, Greece; sarantislivadas@gmail.com; 2 Center for Diabetes and Endocrine Research, University of Toledo College of Medicine and Life Sciences, Toledo, OH 43614, USA; rodis@paparodis.gr; 3 Unit of Reproductive Endocrinology, First Department of Obstetrics and Gynecology, Medical School, Aristotle University of Thessaloniki, 57429 Thessaloniki, Greece; pan.anagnostis@gmail.com; 4 Unit of Endocrinology and Diabetes Prevention and Care, IRCCS Azienda Ospedaliero-Universitaria di Bologna, University of Bologna, 40138 Bologna, Italy; alessandra.gambiner3@unibo.it; 5 Department of Endocrinology, UMC Bežanijska kosa, Faculty of Medicine, University of Belgrade, 11000 Belgrade, Serbia; jbjekic@yahoo.com; 6 Department of Endocrinology, UMC Bežanijska kosa, 11080 Belgrade, Serbia; tijjana91@gmail.com; 7 Division of Endocrinology and Metabolism, Department of Internal Medicine, Hacettepe University School of Medicine, Ankara 06100, Turkey; yildizbo@yahoo.com; 8 Department of Medical Sciences, Serbian Academy of Sciences and Arts, 11000 Belgrade, Serbia; micicd@eunet.rs; 9 Unit of Endocrinology, Diabetes Mellitus and Metabolism, Aretaieion Hospital, Faculty of Medicine, National and Kapodistrian University of Athens, 11528 Athens, Greece; mastorakg@gmail.com; 10 Clinic for Endocrinology, Diabetes and Metabolic Diseases, Faculty of Medicine, University of Belgrade, 11000 Belgrade, Serbia

**Keywords:** PCOS, diabetes mellitus, insulin resistance, androgens, age

## Abstract

Women with polycystic ovary syndrome (PCOS) are at increased risk for dysglycemia and type 2 diabetes compared to healthy BMI-matched women of reproductive age: robust evidence exists supporting this notion. The presence of altered glycemic status in young women with the syndrome presents a distinct challenge for the clinician for several reasons. Firstly, the reported incidence of this disorder varies among the limited available studies. Furthermore, there is a lack of consensus on the best screening method, which women to screen, at what frequency, and which strategies need to be implemented to reduce the above risk. We provide data regarding the prevalence of dysglycemia in young women suffering from PCOS and the pathophysiological mechanisms underlying the disorder. In addition, we present evidence suggesting universal screening with the oral glucose tolerance test in young women with the syndrome, irrespective of age or BMI status, to identify and manage glycemic abnormalities in a timely manner. Regarding follow-up, oral glucose testing should be carried out at regular intervals if there are initial abnormal findings or predisposing factors. Finally, the efficacy of a well-balanced diet in conjunction with regular exercise and the use of non-pharmacologic agents in this specific population is discussed.

## 1. Introduction

Polycystic ovary syndrome (PCOS) is a common endocrine disorder affecting 6–15% of women of reproductive age depending on ethnic variability as well as the criteria used for diagnosis [1]. PCOS presents with a very wide spectrum of clinical features due to its unusually complex pathophysiology. It is a disorder characterized by the intricate interplay between a multitude of factors, incorporating genetic, epigenetic, and environmental stimuli. All these parameters lead to two major etiopathological factors which are believed to lie at the heart of the disorder, namely, androgen excess and insulin resistance (IR) [2].

PCOS is associated with a large number of reproductive and metabolic sequelae, with impaired glucose homeostasis constituting one of the cardinal metabolic features. Indeed, the two prerequisites for type 2 diabetes mellitus (T2DM) development, IR and β-cell dysfunction, are commonly observed in women suffering from PCOS. IR is in fact a fundamental player in PCOS pathophysiology and is further amplified by the presence of obesity [3], while a higher prevalence of pancreatic β-cell dysfunction, associated with increasing age, is observed in women with PCOS compared to their normal peers [4].

Dysglycemia, on the other hand, is thought to occur mainly in older women with PCOS, while there are several as yet largely unclarified issues in younger women suffering from the syndrome [5]. Of note, the incidence of impaired glucose homeostasis as well as the ideal methods for evaluation and management of the disorder in this specific population have also not to date been fully elucidated. It must be mentioned that in this particular age group, androgens are considerably higher than those in women with PCOS older than 35 years of age: as a consequence, amplification of IR is anticipated, which may moreover lead to T2DM development [6]. In this review, we undertake a critical presentation of the available data and shed more light on several areas of uncertainty.

## 2. Pathophysiology of T2DM Development in PCOS

Ovarian androgens are found in higher concentrations in the majority of women with PCOS compared to the general population, a small but significant proportion of these being derived from the adrenal glands [7]. Based on current research, although the exact etiological origins of hyperandrogenemia are not entirely clear, prenatal (in utero) exposure to higher androgen concentrations have been tentatively linked to the syndrome [8]. In addition, higher amplitude of the pulsatile secretion of the gonadotrophin-releasing hormone (GnRH) is seen during puberty in girls affected by PCOS, leading to a more potent excitation of the androgen-producing ovarian cells. This leads to hyperandrogenic symptoms, such as hirsutism, acne, male type hair loss, and ovulatory dysfunction (chronic oligo-anovulation), which produces menstrual irregularity (most commonly oligomenorrhea, i.e., <8 menstrual cycles per year) as well as polycystic ovarian morphology on ultrasound examination, which could lead to infertility in some cases [9].

Even though hyperandrogenemia is the main clinical finding of PCOS in the reproductive years, the metabolic features of the syndrome are equally important. A significant number of teenagers affected by PCOS present with IR, which is in part mediated by genetic predisposition [10]. The disorder is frequently accompanied by pancreatic β-cell dysfunction, hepatic and visceral fat accumulation, increased food intake, and increased waist circumference (central obesity). This, in turn, leads to hyperinsulinemia, which arises due to the inability of the pancreatic islets to enable insulin to exert its actions adequately. The latter is mediated in part by pronounced adipose tissue dysfunction and lipotoxicity frequently found in women with PCOS [11]. Due to this, laboratory findings of this condition could include impaired fasting glucose (IFG), postprandial hyperglycemic excursions (impaired glucose tolerance, IGT), elevations in LDL-cholesterol and triglycerides, lowering of HDL cholesterol, and increased adiponectin and serum markers of inflammation. The sum total of these metabolic derangements can result in the development of T2DM when pancreatic stress reaches a threshold at which insulin production becomes unable to match insulin needs.

IR is an almost universal feature of PCOS, it being found with great frequency, ranging between 44 and 70%, in affected patients [12]. While this finding is more common in obese women with PCOS, it is also often present in their lean counterparts [13]. In adolescents with PCOS, peripheral insulin sensitivity was 50% lower than that found in controls, independent of their body mass index, when measured via hyperinsulinemic euglycemic clamp techniques [14]. IR and β-cell dysfunction are the two prerequisites for development of T2DM both in women with PCOS and in those without the syndrome. The most important trigger of the latter metabolic alteration, however, is obesity. Indeed, in a recent meta-analysis from our group, incorporating data from 319,780 participants (60,336 PCOS and 8847 T2DM cases), a strong interaction between PCOS and development of T2DM was found [RR 3.45 (95% CI, 2.95–4.05, *p* < 0.001)]. Though the risk was higher in the presence of obesity [RR 4.06 (95% CI 2.75–5.98; *p* < 0.001)], it became statistically insignificant in the absence of obesity [RR 2.68 (95% CI 0.97–7.49; *p* = 0.06)] [15].

Nevertheless, there is an enduring argument whether PCOS itself constitutes a risk factor for T2DM or whether T2DM predominantly ensues due to obesity in PCOS [16,17]. A well-designed meta-analysis of genetic studies proposed that PCOS does not possess an inherent risk for T2DM and that, instead, T2DM develops due to elevated androgen levels or as a result of adiposity [18]. Instead, PCOS constitutes a polygenic trait, and sophisticated studies disclosed two different gene clusters. one associated with metabolic disturbances and one related to hyperandrogenic signs [19]. Therefore, a genetic component of dysglycemia among PCOS women should be considered.

Dysglycemia, which is an imbalance in the body’s ability to maintain blood sugar levels, is one of the most characteristic metabolic abnormalities in PCOS and should be considered as a continuum, progressing from normoglycemia to impaired fasting glucose (IFG) and/or impaired glucose tolerance (IGT) and overt T2DM. However, it should be noted that IFG is mostly detected in subjects with mostly hepatic IR and normal muscle insulin sensitivity. On the contrary, severe muscle IR accompanied with normal liver insulin sensitivity is found in those subjects with isolated IGT [20]. This observation is of major importance given that IR in women with PCOS is amplified by androgens and vice versa. Indeed, progression of T2DM is significantly higher in hyperandrogenic women with PCOS [21]. In fact, molecular studies in lean women with PCOS have demonstrated that androgens reduce muscle insulin sensitivity, providing a possible explanation for this phenomenon [22]. However, cross-sectional data have reported a gradual decrease of androgens in PCOS and controls [23] through time, followed by a parallel reduction of IR in lean women with PCOS [24,25]. Therefore, one may speculate that women with IFG may be prone to T2DM development, whereas the IGT group may possibly include individuals who experience dysglycemia due to hyperandrogenemia. Of interest, in our study assessing 1614 Caucasian women with PCOS, we found that the degree of hyperandrogenemia was significantly higher in women with IGT compared to those with either IFG or T2DM, implying a different impact of androgens in this specific group [26].

We therefore suggest that these women may not develop T2DM over the years if they remain lean, highlighting the importance of normal-weight maintenance in women with PCOS. This notion is of major importance given that normal-weight women constitute about 25% of women with PCOS [27] and that the spectrum of clinical and laboratory findings is dominated by obese and overweight women, who form the main body in PCOS cohorts. Otherwise expressed, the incidence of PCOS is 28% in obese and overweight women and only 5.5% in women of normal weight, emphasizing the impact of extreme adiposity in PCOS pathophysiology [28]. In a detailed meta-analysis, it was shown that higher BMI exacerbated insulin sensitivity reduction by 15% compared to the patients’ lean counterparts [13], which can overcome compensatory insulin secretion, leading to dysglycemia.

## 3. T2DM in Adolescents with PCOS

### Methods

The incidence and methods of dysglycemia assessment in young women with PCOS were determined.

We searched MEDLINE, SCOPUS, and COCHRANE using the following selection criteria: (“Polycystic Ovary Syndrome”[MeSH] OR “Amenorrhea”[MeSH] OR “polycystic ovary syndrome”[tiab] OR “polycystic ovarian syndrome”[tiab] OR PCO[tiab] PCOS[tiab] OR “Stein-Leventhal Syndrome”[tiab] OR “Stein Leventhal Syndrome”[tiab] OR oligomenorrhea[tiab] OR oligomenorrhoea[tiab] OR amenorrhea[tiab] OR amenorrhoea OR oligoamenorrhea[tiab] OR oligo-amenorrhea[tiab] OR oligoamenorrhoea[tiab] OR oligo-amenorrhoea[tiab] OR anovulat*[tiab] OR oligoanovulat*[tiab] OR oligo-anovulat*[tiab] OR hyperandrogenemia[tiab] OR hyperandrogenaemia[tiab] OR androgenism[tiab] OR hyperandrogenism[tiab]) AND (“diabetes mellitus, Type 2”[MeSH] OR (diabet*[tiab] AND (“non-insulin dependent”[tiab] OR “non-insulin-dependent”[tiab] OR type-2[tiab] OR “type 2”[tiab] OR “type II”[tiab]))) NOT (Animal[MeSH] NOT Human[MeSH]) NOT (letter[pt] OR comment[pt] OR editorial[pt] OR Review[pt] OR “practice guideline”[ptyp] OR “case reports”[ptyp]).

First, abstracts in English from papers published from 1995 to 2022 were assessed to evaluate their relevance to the combined clinical effect of the two conditions, psychosis and PCOS. The final inclusion/exclusion of articles was not based on a protocol developed before as in a systematic review. Only observational studies were included with age less than 30 years, and the subjects studied were more than 65 women. The primary outcome measures were IFG, IGT, and TDM2.Two review authors independently selected the studies, extracted the data according to the protocol, and assessed study quality. We assessed the overall quality of the evidence using the GRADE approach.

**Figure 1:**
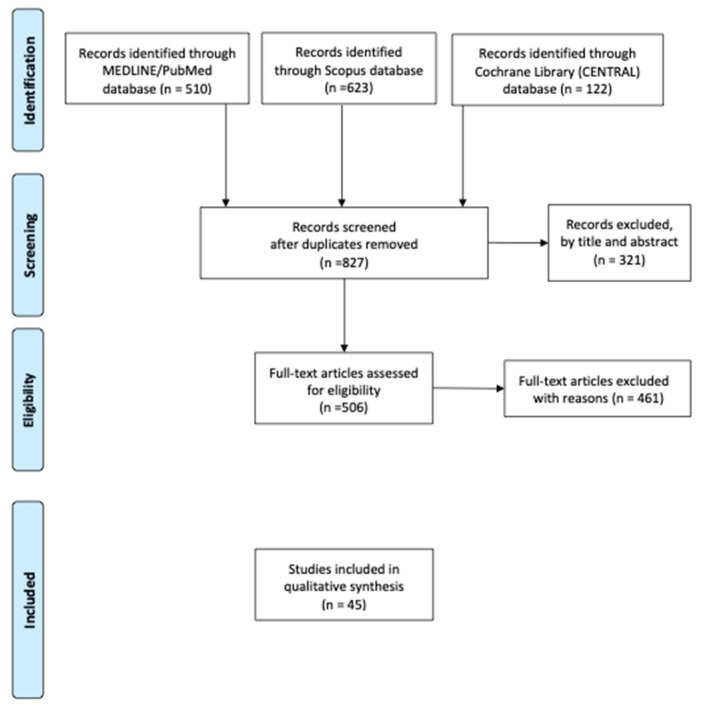
Literature review flow chart.

The chart flow is shown below in Figure 1.

The incidence of PCOS in adolescents is significantly lower than in those older than 20 years, as was revealed by applying the NIH criteria in a cohort of 137,502 adolescents aged 15 to 19 years. The actual incidence was 1%, and obesity was highly associated with PCOS diagnosis [29]. Therefore, it is obvious that despite the ample evidence regarding the relationship of PCOS and T2DM in adults, very few data exist concerning affected adolescents. A recent systematic review and meta-analysis of published studies performed a reverse analysis to assess the prevalence of PCOS in adolescents with T2DM: it identified an impressive 24.04% (95% CI, 15.07–33.01%; I2 = 0%; *p* = 0.92) [30].

On the other hand, the largest study to date assessing the effects of PCOS on the risk of T2D development in adolescents included data from 493 obese girls with PCOS followed up for approximately 2 years, leading to 23 incident cases of T2DM [31]. In this study, the reported incidence rate of T2DM in obese girls with PCOS was 22.6 cases per 1000 patient-years (PYs) of follow-up, being much higher than the 0.16 cases per 1000 PYs reported for the general, age-matched but not BMI-matched population [32], or the 4.7–8.8 cases per 1000 PYs reported for the adult PCOS population [33]. According to this study, a metanalysis of 16 studies comparing adolescents with PCOS (obese vs. lean) highlighted the impact of obesity. In fact, in obese subjects, a worse metabolic profile, including higher triglycerides, leptin, fasting insulin, LDL-C, and free testosterone levels, was found [34], and moreover, cardiovascular risk factors and subclinical atherosclerosis were reported in obese adolescents suffering from the syndrome [35].

Such inferential comparisons do not, of course, provide proof of a significantly higher risk of development of T2DM in adolescent patients with PCOS as compared to their adult counterparts, because no studies directly comparing these groups of patients exist. However, one recent study from Brazil evaluating 62 adolescents and 248 adult women with PCOS reported that most biomarkers of glucose metabolism abnormalities were similar in adolescents and adults with PCOS [36]. Clearly, appropriately designed studies are required to address these questions.

## 4. Prevalence and Methods of Assessment of Dysglycemia in Women with PCOS

In general, the prevalence of dysglycemia, including T2D, IGT, and IFG, is higher in women with PCOS compared to healthy BMI-matched women of reproductive age: namely, in PCOS it ranges from 1.5 to 12.4%, while in normal women of reproductive age, it is 1–3% [37]. With regard to young women with PCOS, the prevalence of these conditions ranges from 2–14.5% for IFG, from 5.9 to 34% for IGT, and from 1.5–10% for IFG, as illustrated in Table 1. However, the above numbers are extrapolated from the literature data, since the vast majority of available studies provide no strict classification according to age. In addition, there is significant variability among the available data due to the different definitions applied for IFG status (the American Diabetes Association (ADA) or the World Health Organization (WHO) criteria) and the PCOS criteria used. The reality is that a higher degree of dysglycemia is expected in women diagnosed with the more strict NIH criteria compared to the mild phenotype D of the Rotterdam criteria (namely, the coexistence of ovulatory dysfunction and polycystic ovaries on ultrasound) due to the lower grade of IR demonstrated in this subgroup [38]. However, this hypothesis was not corroborated in a large study analyzing data of 2000 women wherein a similar T2DM prevalence was documented among different PCOS phenotypes [39]. The considerable heterogeneity observed could, furthermore, be partly due to the wide range of different countries and races discussed in the studies. In a large cross-sectional study carried out by our group of 628 women with PCOS aged from 17 to 25 years, while we found that according to the ADA criteria the incidence of IFG was 35%, this percentage was lower by one-third (11%) based on the WHO criteria. IGT and T2DM were observed in 7.5% and 1.1% of this subgroup, respectively. Of interest, a similar pattern was demonstrated in the other two subgroups, namely, in 650 women aged 26 to 35 years of age and in 336 women older than 35 years [26].

The Wilson and Jungner criteria suggest screening for a disease when it poses a significant health issue, an established therapeutic approach for this disorder is available, facilities for diagnosis and treatment exist, and other prerequisites are met [40]. Accordingly, it is evident that screening for T2DM in women with PCOS constitutes a mandatory step for the evaluation of women with the syndrome. However, the optimal method for assessment of glycemic status has not been agreed upon to date and there is no consensus on which women to screen.

The available tools for glycemic status evaluation are fasting plasma glucose (FPG), the oral glucose tolerance test (OGTT), and glycated hemoglobin (HbA1C). The implementation of HbA1C is not considered appropriate in PCOS for several reasons. Mainly, due to menstrual irregularities, periods of oligomenorrhea are often followed by periods of heavy bleeding, which significantly affects hemoglobin levels and consequently modifies HbA1C values [41]. Furthermore, in two studies in women with PCOS, it was not well correlated with OGTT findings [42,43]. Moreover, recent data have questioned the role of HbA1c in the diagnosis of dysglycemia in overweight and obese subjects, who represent almost 75% of the PCOS population [44]. Finally, it is important to note that HbA1c testing is a costly procedure, while there is still significant variation in the way it is carried out globally. Additionally, there is variation in the results of HbA1c testing across different ethnicities, which makes it difficult to standardize the test for everyone. The lack of international standardization of HbA1c testing further complicates matters. In addition, the implementation of HbA1c as a marker of dysglycemia is also weakened by the disagreement between the diagnostic criteria for prediabetes suggested by the WHO and those proposed by the ADA. The diagnostic threshold for HbA1c as a marker of prediabetes is 42 mmol/mol (6.0%) according to the WHO, while it is 39 mmol/mol (5.7%) according to the ADA. This discrepancy can lead to confusion in diagnosis and management of prediabetes and diabetes.

On the other hand, despite being more complicated, costly, and time consuming than other available methods, OGTT is considered the gold standard for T2DM diagnosis because it is standardized and can detect IGT, which is important for women with PCOS. Early detection of IGT in this at-risk population can lead to lifestyle modifications and/or pharmacological intervention that may prevent or delay the development of T2DM [45]. Furthermore, the applicability of OGTT in patients with iron deficiency usually encountered in these women and the possibility of parallel evaluation of insulin levels post glycemic load as an accurate estimate of the degree of IR are two more benefits of this procedure [46].

Finally, both the ADA and the WHO are in agreement as regards the glucose level cut-off for the diagnosis of IGT [47,48]. There is a significant amount of evidence that an isolated FPG could categorize a noteworthy number of subjects with either IGT or T2DM as having normal glucose homeostasis. This misclassification rate ranges from 20–40%, which is not negligible given the increased risk for T2DM in women with PCOS from their early reproductive years [49,50,51]. Furthermore it is important to consider that there is a 3–6% variation in most commercially available glucose assays, which can easily lead to the characterization of the same patient as having either normal glucose tolerance or IFG. Therefore, when interpreting glucose results, clinicians should be aware of this potential source of error and take into account the specific assay used. In cases where the diagnosis of IFG is in doubt, it may be appropriate to repeat the fasting glucose measurement or perform an OGTT to confirm the diagnosis.

From all the above, it is obvious that OGTT allows for the detection of abnormalities in glucose metabolism that may not be apparent with a single measurement of FPG, making it a valuable tool for identifying individuals at high risk for T2DM, while it is a more accurate index for the assessment of glycemic status in all women suffering from the syndrome [26].

**Table 1:** Prevalence of dysglycemia in young women with PCOS.

Group	Year	*n*	Country	PCOS Criteria	T2DM Criteria	Age (Years)	BMI (kg/m^2^)	IFG (%)	IGT (%)	T2DM (%)
Rajkhowa et al. [52]	1996	90	UK	N	W	26	31	?	9	2
Legro et al. [53]	1999	254	USA	N	W	14–44	32 ± 3	?	31	7.5
Ehrmann et al. [54]	1999	122	USA	N	A	25 ± 0.7	30–43	9	35	10
Gambineri et al. [55]	2004	121	Italy	R	W	14–37	20–38	?	15.7	2.5
Chen et al. [56]	2006	102	China	R	W	24.2 ± 6	21.7 ± 4	?	20.5	1.9
Mohlig et al. [50]	2006	264	Germany	N	W	28 ± 0.4	30 ± 0.4	?	14.3	1.5
Vrbikova et al. [57]	2007	244	Czech	R	A	27 ± 7.5	27 ± 6.9	12.3	9.4	1.6
Espinos-Gomez al. [58]	2008	102	Spain	N	W	26 ± 6	30.2 ± 8	?	10.7	7.7
Bhattacharya et al. [59]	2009	264	India	R	W	24 ± 4	27 ± 4.5	?	14.4	
Zhao et al. [60]	2010	818	China	R	A	25 ± 5	?	8.5	35.4	4
Stovall et al. [61]	2011	78	USA	N	A	26 ± 6.4	29 ± 6	2	14	?
Celik et al. [43]	2013	252	Turkey	R	A	24 ± 5	26 ± 5.7	?	14.3	2
Lerchbaum et al. [62]	2014	714	Austria	R	A	27 (23–32)	24.2	12.8		1.5
Ganie et al. [63]	2015	2014	India	R	A	23 ± 5.4	25 ± 4.4	14.5	5.9	6.3
Li et al. [64]	2016	2436	China	R	A	27	21.56	13.5	19.8	3.9
Pelanis et al. [65]	2017	876	Sweden	R	A	29 (25–34)	28 (23–33)	11	12	3
Zhang et al. [39]	2018	378	China	R	IDF	27 ± 4.4	30 ± 4.3	31.5		8.7
Ortiz-Flores et al. [66]	2019	400	Spain	R	W	26 (14–49)	28.6	14	14.5	2.5
Choi et al. [67]	2021	262	Korea	R	A	23 ± 5.7	22.7 ± 4.2	19.5%		1.6%

N: NIH, R: Rotterdam, W: WHO, A: ADA. “?” means lack of data.

## 5. Baseline Screening in Young Women with PCOS

The question of whether glycemic status should be evaluated in every woman with PCOS or only in certain subgroups remains thus far unanswered. Two points of view exist regarding who should be screened via the OGTT (Table 2). One view, supported by the Endocrine Society, the Androgen Excess and Polycystic Ovary Syndrome Society (AE-PCOS), and Australian guidelines, suggests universal screening for all women with PCOS [68,69,70]. The other view favored by the European Society of Human Reproduction and Embryology and the American Society of Reproductive Medicine recommends screening for women with a minimum of one risk factor, such as age over 40 years, a family history of type 2 diabetes or gestational diabetes mellitus, or obesity [71,72,73].

Nevertheless, this recommendation has not been substantiated by solid data, as studies have argued both for and against it [53,74]. Notably, there are strong supportive data for the criterion of a family history of type 2 diabetes in studies from the USA and Australia [54,75] but not in studies originated from European countries [17,50]. Therefore, while these criteria seem reasonable, they may not reflect the different course of T2DM development in women with PCOS compared to that in the unaffected population. Additionally, most studies that endorse these recommendations did not assess the effect of age, obesity, and hyperandrogenemia on the progress of carbohydrate disorders, which further complicates the issue [74,76]. Clearly, more research is needed to determine the best approach to glycemic screening in women with PCOS. Particularly regarding young women, half of the patients with T2DM and 70% of those with IGT would not have been correctly diagnosed with the use of FPG in our cohort of 625 women aged 17 to 25 years [26]. Therefore, we are strongly in favor of OGTT in baseline screening of every young woman with PCOS.

## 6. Evolution to T2DM and Frequency of Glycemic Status Assessment

The progress from normal glucose levels to IGT or from IGT to T2DM in women with PCOS has been calculated to be lower than that in the general population. In fact, in individuals with IGT among the general population, the conversion rate to T2DM is estimated at 7% annually [77], which is significantly higher than the analogous annual progression rate from 2.5 to 3.6% observed in PCOS [78,79,80]. Therefore, one could speculate that the primary mechanisms of T2DM development in PCOS are different from those found in the healthy population. Of note, non-linear progression to T2DM is common in PCOS, related to BMI [21]. Obese women exhibit a four-fold greater risk of developing IGT, while lean women with PCOS have the same probability to develop T2DM as controls. In particular, Celik et al. found that obese women with PCOS were at a four-fold greater risk of conversion from normal glucose tolerance to IGT compared to lean women with PCOS, further supporting the correlation between adiposity and risk of progression to T2D in PCOS [81]. Rubin et al. also reported that normal-weight women displayed a similar risk of progressing to T2D to that of controls, which suggests that the presence of PCOS alone may not increase the risk of T2D in lean women [74]. This notion should be attributed to the different evolution of IR in PCOS, as available data have shown improvement in lean women and deterioration in obese, whereas in controls a gradual deterioration of IR is anticipated [24,25].

Hence, the frequency of glycemic status assessment in PCOS women is a topic of debate among experts, with recommendations ranging from yearly to every 5 years depending on additional factors (Table 3). Specifically, the Endocrine Society recommends performing an OGTT every 3–5 years or more frequently if risk factors are present, while the PCOS Special Interest Group of the European Society of Endocrinology recommends performing an OGTT in all obese patients with PCOS and in lean middle-aged patients with additional risk factors. Regarding young women, we suggest that the frequency of glycemic status assessment should be individualized based on the patient’s risk factors, such as family history of T2DM, obesity, and history of gestational diabetes, as well as the presence of symptoms suggestive of T2DM or substantial weight gain. An OGTT is the preferred method for diagnosing impaired glucose tolerance and T2DM in PCOS patients, while fasting glucose levels and HbA1c have limited sensitivity in identifying prediabetes. In addition, regular monitoring with OGTT can also help clinicians to adjust treatment and lifestyle interventions as needed to prevent or delay progression to T2DM in women with PCOS.

## 7. Strategies to Minimize Diabetes Risk in Young Women with PCOS

There are different clinical possibilities for the general reduction of metabolic risk, while diabetes risk may be minimized using diagnostic tools as well. Whereas in overt T2DM, fasting glucose could represent an initial risk marker for IGT and T2DM, this notion is questioned in the case of women with PCOS. Recent analyses of a large Europid cohort of PCOS women reported that one-third of those with IGT or T2DM had normal basal glucose [26]. As discussed above, OGTT gives evidence of being a relevant tool for the assessment of diabetes risk that may be periodically used for the evaluation of glycemic status in all women with PCOS, including young patients [82].

In current clinical practice, women with PCOS are often informed as to the possibility of their developing diabetes during the course of their life, while patients with PCOS, especially those of older age, are aware of and concerned about future metabolic derangements and, specifically, T2DM development [83]. In this context, a few small studies have reported the encouragingly positive outcomes of lifestyle interventions, including a dietary regimen combined with physical activity even over a short period. Meanwhile, lifestyle intervention including 1200–1400 kcal/day diet during 6 months, followed by mild dietary restriction combined with regular exercise during the rest of the follow-up period of about 2 years resulted in complete resolution of both hyperandrogenism and menstrual irregularity in 36% of the PCOS women [84]. However, because the benefits of physical activity in ameliorating reproductive dysfunction in PCOS are less well established, a clearer definition of the exact type, frequency, and duration of exercise for this particular purpose is needed [85]. Another well-established management practice for PCOS patients is lifestyle modification accompanied by metformin therapy, a combination that today is approved for the treatment of both prediabetic and PCOS patients [86]. As to PCOS treatment comprising pharmacological management in addition to lifestyle intervention, this approach is recommended for diabetes prevention in PCOS patients at the highest risk [18].

Alpha-lipoic acid (ALA) is a natural compound of therapeutic use for the amelioration of IR. ALA has been shown to be more effective in the prevention of glucose metabolism alterations rather than in their treatment. Although numerous small clinical studies in PCOS women have been conducted using ALA either alone or in combination with other insulin-sensing agents such as inositols [87], only one study can be considered valid due to the use of the euglycemic hyperinsulinemic clamp technique. Administration of 1200 mg/day of ALA in a small group of lean PCOS women led to a significant improvement in insulin sensitivity: this indicated a possible favorable effect on the patients’ menstrual cycles, without, however, achieving any change in body weight. Of note, it was suggested that the effects of ALA may be mediated through a mechanism not necessarily involving oxidative stress changes [88].

Vitamin D (VD) deficiency has been shown to be associated with many signs present in PCOS, including adiposity, inflammation, insulin resistance, and diabetes [89]. Therapeutically, VD supplementation as an add-on therapy can lead to an improvement in IR and lipid metabolism, a reduction in circulating androgens, as well as a better response to ovulation induction in PCOS women [90]. It is hypothesized that these effects are mediated through improvement in high-sensitivity C-reactive protein and total antioxidant capacity in PCOS women on VD supplementation [91].

The potential for the use of genetic data in personalized medicine in PCOS patients has recently been suggested. In addition, overweight and obese PCOS women with hyperandrogenemia may benefit considerably from a diabetes prevention program. On the other hand, normal-weight PCOS women with normal androgen concentrations do not need to be stressed by being classified as a risk group for diabetes, since no association has been found between genetically predicted PCOS and risk of diabetes. Accordingly, normal-weight PCOS women should be counseled to avoid weight gain which could confer this risk [18].

In conclusion, given that PCOS is associated with an increased risk of dysglycemia and T2DM, screening for these conditions is recommended in young women with the syndrome. However, there is still uncertainty regarding the optimal screening strategy as well as the exact mechanisms underlying progression of dysglycemia in PCOS. In our opinion, diagnosis and follow-up should be based on the OGTT, while weight management, regular exercise, and a well-balanced diet should be emphasized among all patients. Further research is needed to better understand these issues and to develop evidence-based guidelines for the management of glycemic abnormalities in young women with PCOS.

## Figures and Tables

**Table 2:** Guidelines regarding OGTT upon diagnosis in all women with PCOS.

Body	Suggestion
Joint AACE/ACE and AE-PCOS society	Yes
Australian NHMRC	No (Recommended if: BMI > 25 kg/m^2^—iAsians > 23 kg/m^2^, history IFG, IGT, GDM, family history of T2DM, hypertension or high-risk ethnicity)
Endocrine Society	Yes
Royal College of Obstetricians & Gynecology	No (Recommended if one or more: BMI ≥ 25 kg/m^2^, age ≥ 40 years, previous gestational diabetes or family history of T2DM)
AE-PCOS Society	No
ESHRE and ASRM	No (Recommended if BMI ≥ 27 kg/m^2^)

**Table 3:** Guidelines regarding follow-up OGTT.

Body	Suggestion
Joint AACE/ACE and AE-PCOS society	Yearly in women with IGT Every 1–2 years, based on BMI (not specified) and family history of T2DM
Australian NHMRC	Every 1–3 years, based on presence of other diabetes risk factors
Endocrine Society	Every 3–5 years. Sooner if additional risk factors for T2D
Royal College of Obstetricians & Gynecology	Annually in women with IGT or IFG
AE-PCOS Society	Every 2 years in women with risk factors Sooner if additional risk factors for T2D develop
ESHRE and ASRM	Not specified

## Data Availability

Data sharing not applicable.
